# Periconception Weight Loss: Common Sense for Mothers, but What about for Babies?

**DOI:** 10.1155/2014/204295

**Published:** 2014-04-02

**Authors:** Kristine Matusiak, Helen L. Barrett, Leonie K. Callaway, Marloes Dekker Nitert

**Affiliations:** ^1^School of Medicine, The University of Queensland, 288 Herston Road, Herston, QLD 4006, Australia; ^2^The UQ Centre for Clinical Research, The University of Queensland, RBWH Campus, Butterfield Street, Herston, QLD 4029, Australia; ^3^The Royal Brisbane and Women's Hospital, Butterfield Street, Herston, QLD 4029, Australia

## Abstract

Obesity in the childbearing population is increasingly common. Obesity is associated with increased risk for a number of maternal and neonatal pregnancy complications. Some of these complications, such as gestational diabetes, are risk factors for long-term disease in both mother and baby. While clinical practice guidelines advocate for healthy weight prior to pregnancy, there is not a clear directive for achieving healthy weight before conception. There are known benefits to even moderate weight loss prior to pregnancy, but there are potential adverse effects of restricted nutrition during the periconceptional period. Epidemiological and animal studies point to differences in offspring conceived during a time of maternal nutritional restriction. These include changes in hypothalamic-pituitary-adrenal axis function, body composition, glucose metabolism, and cardiovascular function. The periconceptional period is therefore believed to play an important role in programming offspring physiological function and is sensitive to nutritional insult. This review summarizes the evidence to date for offspring programming as a result of maternal periconception weight loss. Further research is needed in humans to clearly identify benefits and potential risks of losing weight in the months before conceiving. This may then inform us of clinical practice guidelines for optimal approaches to achieving a healthy weight before pregnancy.

## 1. Introduction

Obesity in pregnancy is increasingly common in developed nations [1–3]. This is a growing concern as maternal obesity is a risk factor for a number of adverse maternal and offspring outcomes [1, 4–12]. Current consensus guidelines advocate for healthy body mass index (BMI) prior to pregnancy [13–15], but there is no agreed-upon strategy for women to achieve healthy weight before conceiving. There is evidence for improved pregnancy outcomes following weight loss between pregnancies [7, 16, 17]. Whether negative energy balance during the periconceptional period has disadvantages must also be considered. Insults to the reproductive milieu are likely to have both immediate and long-term consequences for a developing organism.

There is well-established animal research which focuses on the topic of periconceptional undernutrition that illuminates these potential risks to offspring. Corroborating this are data from cohorts of people conceived in the Dutch Famine during World War II and in seasonal famines in The Gambia suggestive of long-term health consequences. With the exception of these epidemiological studies from the Dutch Famine, human studies examining offspring outcomes following periconceptional undernutrition are lacking. It is important to determine whether benefits of weight loss during this period are in conflict with potential risks to the long-term health of offspring. Clarifying the critical windows of early development may then lead to the development of evidence-based guidelines for safe weight management in the obese obstetric population, with long-term offspring health in mind.

## 2. Obesity and Pregnancy: Scope of the Problem

Overweight and obesity are increasingly common in the obstetric population, affecting up to one-third of childbearing women in developed countries [2, 3, 13]. Maternal obesity is a major independent risk factor for numerous pregnancy and neonatal complications, some of which predispose the baby to disease in adulthood. From an economic standpoint, maternal obesity is associated with longer hospital stays and therefore greater costs compared to normal weight women [18, 19]. There is therefore growing interest in the management of obesity as a means of preventing these complications through lifestyle modification in the time prior to pregnancy.

## 3. Obesity and Pregnancy: Adverse Outcomes Associated with Overnutrition 

The consequences of obesity for reproductive health range from reduced fertility to increased incidence of gestational disease and adverse neonatal outcomes.

### 3.1. Maternal Complications

Obesity is associated with reduced fertility [43] and is an independent risk factor for spontaneous pregnancy termination among women using infertility treatment [4, 12]. Well-documented risks of obesity in pregnancy include gestational diabetes, hypertension, preeclampsia, thromboembolism, perinatal infection, and caesarean section [1, 2, 5, 44–46]. A large population-based study of interpregnancy weight change was the first to provide evidence for a causal relationship between higher BMI and adverse outcomes [11]. Relatively small increases in weight between first and second pregnancies were associated with greater complications [11].

Some pregnancy complications associated with obesity are themselves risk factors for subsequent disease in mothers. Women who have had gestational diabetes incur at least a sevenfold increased risk of type 2 diabetes [47] and preeclampsia is associated with subsequent risk of cardiovascular disease [48].

### 3.2. Fetal/Neonatal Complications

Neonatal morbidity associated with maternal obesity includes hypoglycemia, respiratory distress, macrosomia, birth defects, and greater rate of admission to neonatal intensive care [2, 5, 49]. Preterm birth, stillbirth, and neonatal mortality are also more frequent [1, 8, 10, 44]. Overweight and obesity are the greatest modifiable risk factors for stillbirth in high-income countries [6].

Several of the complications associated with maternal obesity are not only a risk to health at the time of birth but are also risk factors for adult disease in the offspring [50]. Macrosomia and gestational diabetes are both examples of complications that are then risk factors for obesity and type 2 diabetes mellitus later in life [51].

Mechanisms for how obesity predisposes to adverse outcomes are not yet clearly understood. Developmental pathways likely to be involved include central mechanisms regulating appetite and peripheral mechanisms involving insulin sensitivity and cardiovascular regulation [9]. On the molecular level, oxidative stress, inflammation, and epigenetic modifications may be operating in the propagation of metabolic disease from mother to child [9]. Specifically, altered offspring hypothalamic-pituitary-adrenal (HPA) axis function has been implicated in programming the metabolic syndrome in offspring [52].

## 4. Obesity and Pregnancy: Current Approach to Management

Given the evidence for poorer pregnancy outcomes associated with obesity, current National Institute for Health and Care Excellence (NICE), American Congress of Obstetricians and Gynecologists (ACOG), and the Royal Australian and New Zealand College of Obstetricians and Gynaecologists (RANZCOG) guidelines advocate for a BMI within the normal range prior to falling pregnant [13–15]. The guidelines also call for health professionals to educate patients about associated harms of obesity and pregnancy and to actively counsel or implement weight loss programs using evidence based strategies before their patients conceive [13, 14]. While clinicians and patients may be aware of obesity risks in pregnancy, there is no evidence-based strategy to guide preconception weight loss or the timing of that weight loss. General recommendations include strategies of dietary modification and exercise but do not prescribe a period of intervention or rate of weight loss that is most compatible with reproductive health. As the process of follicle maturation for a given menstrual cycle begins months before ovulation, weight loss may have implications for pregnancy well in advance of conception. It is therefore necessary to determine the potential benefits and risks associated with maternal weight change during this time and the mechanisms by which these occur.

## 5. Preconception Weight Loss: Current Evidence in Humans

Weight loss as a means for improving health in overweight and obesity has been widely validated, but there are currently no randomized controlled trials evaluating pregnancy outcomes after weight loss prior to pregnancy. A population-based study showed that a moderate reduction (4.5 kg) in prepregnancy weight is associated with lower risk of gestational diabetes [7]. Other studies of interpregnancy weight loss demonstrate reduction in risk for gestational diabetes and preeclampsia [16, 53]. Bariatric surgery has been shown to improve pregnancy outcomes in obese women [54–56]. Weight loss surgery has also been shown to result in lower rates of obesity, greater insulin sensitivity, and an improved lipid profile among children born after weight loss surgery compared to their siblings born before [57, 58]. This improvement in cardiometabolic profile is potentially attributed to differential methylation of a subset of glucoregulatory and inflammatory pathway genes in the after-surgery offspring [59, 60].

The period of maximal weight loss following Roux-en-Y procedures is typically 12–18 months, after which weight plateaus. Retrospective studies have found no difference in rates of obstetrical complications between those who conceive during this period of weight loss and those who conceive after this period [61, 62]. Long-term followup of offspring conceived during this weight loss period is lacking; however, there is an ongoing recommendation to exercise caution if falling pregnant in the early postoperative period, however evidence from long-term follow up of offspring conceived during this weight loss period is lacking.

## 6. Preconception Weight Loss: Potential Disadvantages

In order to confidently advocate for weight loss prior to pregnancy, the health profession must consider the potential risks associated with this behaviour change. Up to half of pregnancies are unplanned [63] and many women are unaware that they are pregnant for the first several weeks of gestation. For this reason, studies examining nutritional influences on all stages of the periconceptional period, including early gestation, are relevant to women attempting weight loss for pregnancy.

“Periconception” refers to “developmental stages which include some or all of the following early events: oocyte maturation, follicular development, conception, and embryo/blastocyst growth up until implantation” [64]. The periconceptional period is characterised by prolific cell division but has relatively low energy demands. It is therefore important to determine the potential influence of energy restriction during this period and to further elucidate the mechanisms by which dietary restriction may impact the maternal-fetal system. The weeks following implantation, early gestation, also represent an impressionable period in development.

## 7. Epidemiological Data: The Dutch Famine and Seasonal Undernutrition in Gambia

Epidemiological data from the Dutch Famine provide insight into potentially life-long consequences of undernutrition in the periconceptional and early gestational periods (Table 1). This 5-month famine during the Second World War was characterized by severely restricted food rations in The Netherlands, varying between 400 and 800 daily kilocalories at its extreme. Cohorts conceived or in utero during this famine provide evidence for differential effects of the timing of maternal undernutrition. Subjects exposed to the famine around conception or early gestation are more likely to experience a range of adult disease, largely distinct from those exposed at other points of gestation. Higher rates of glucose intolerance, atherogenic lipid profile, altered coagulation profile, obesity in women, altered stress sensitivity and response, coronary heart disease, schizophrenia, addiction, and breast cancer are observed in this cohort [65–77]. Importantly, many of these adult outcomes are independent of size at birth. The cohort exposed to famine in mid and late gestation was smaller at birth and had reduced glucose tolerance and higher rates of obstructive airways disease and microalbuminuria [65, 75, 76, 78, 79]. In addition, there may be a birth weight effect on offspring of people conceived during the Dutch Famine, lending support to epigenetic modification as a potential compensation for undernutrition [80]. Those people exposed to famine specifically during the periconception period demonstrate in adulthood persistent hypomethylation of the* IGF* gene compared with their unexposed siblings of the same sex [81].

Interesting patterns relating to maternal undernutrition and gestational length have also been reported from The Gambia [82, 83] (Table 1). Pregnancies conceived in the rainy “hungry” season (from September to November) where maternal weight and fat stores drop significantly are significantly shorter than those that were conceived in the dry season. Individuals born in the rainy season have an overall increased chance of death in young adulthood which may be associated with changes to the development of the immune system [84]. Individuals conceived in the rainy season had higher DNA methylation levels of metastatic epialleles, providing a mechanism through which the effects of maternal nutrition state at conception are conveyed [85]. Patterns of low birth weight, however, mirrored maternal weight, with the highest incidence in the rainy season.

## 8. Animal Studies

**Figure 1:**
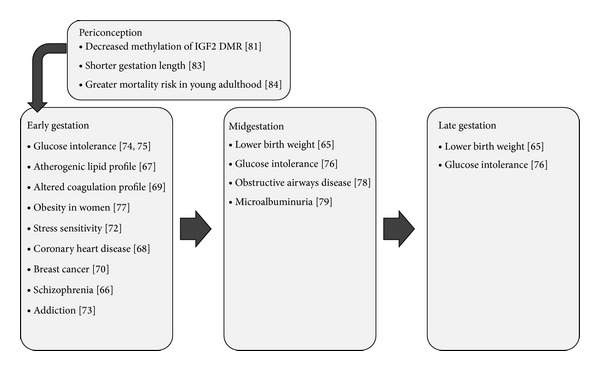
Conditions found at higher rates among cohorts exposed to maternal undernutrition during different periods of gestation (Dutch Famine and Gambian studies).

Research on animals, primarily ewes, provides support for many of the findings in epidemiological studies and highlights additional consequences of maternal undernutrition (Figure 1). Some of this research has confirmed advantages to weight loss in obese animals. Feeding obese ewes a moderately restricted diet (30% general caloric restriction), reduces female offspring fat mass typically associated with maternal obesity to control levels in a model where the embryos were transferred to nonobese ewes at six to seven days after conception [32]. There are, however, potential adverse immediate and long-term changes in offspring exposed to periconceptional undernutrition involving endocrine, developmental and cardiovascular systems.

### 8.1. Periconceptional Undernutrition and the Hypothalamic-Pituitary-Adrenal (HPA) Axis

Periconceptional undernutrition is associated with changes in offspring HPA axis function from early gestation to adulthood. The programming and subsequent function of the HPA axis has broad implications for health, as disturbed HPA axis function is associated with obesity and cardiovascular-related mortality [86, 87].

#### 8.1.1. Early HPA Axis Activation and Preterm Birth

Activation of the HPA axis in sheep is essential for initiating parturition [88]. Maternal undernutrition in the periconceptional period is associated with earlier activation of the HPA axis in late gestation [22, 23]. The molecular mechanism by which this occurs is unclear, as measured mediators of steroidogenesis, MC2R and StAR mRNA, are not correlated with adrenocorticotropic hormone (ACTH) in late gestation in the periconceptional undernutrition offspring [89]. Not surprisingly, periconceptional undernutrition is also associated with preterm birth [24, 25].

#### 8.1.2. Altered Exposure to Maternal Glucocorticoids

Abnormal exposure to maternal glucocorticoids potentially explains altered fetal HPA axis function. In the same study that reported earlier activation of fetal HPA axis following periconceptional undernutrition, maternal ACTH and cortisol levels were suppressed during undernutrition and for several weeks following refeeding [23]. Placental isoforms of hydroxyl-steroid dehydrogenase (HSD) are important gatekeepers in modulating fetal exposure to maternal steroids, as they convert cortisol to cortisone. In midgestation, placental 11-beta-HSD2 isozyme activity is suppressed with an associated fetal elevation in cortisol to cortisone ratio [20]. Suppression of 11-beta-HSD2 allows greater fetal exposure to active stress hormone from maternal circulation. In late gestation, however, placental 11betaHSD activity in periconceptionally undernourished fetuses does not appear to be different from that of controls, and the cortisol : cortisone ratio is significantly lower [21]. The fetal adrenal gland is relatively inactive during midgestation; thus the placenta is likely to be the main mediator of fetal HPA axis activation via controlled exposure to active maternal cortisol [90]. Altered maternal HPA axis function as well as dysfunctional placental modulation may therefore contribute to or work in series with other mechanisms to stimulate the earlier activation of the HPA axis in periconceptionally restricted offspring.

#### 8.1.3. Altered Postnatal HPA Axis Function and the Role of Epigenetics

Functional differences in the HPA axis persist postnatally following exposure to undernutrition around conception and early gestation, despite adequate nutrition throughout the remainder of gestation. There are variable findings reflecting different methods of undernutrition, although there consistently appears to be a difference in HPA axis function from controls. In offspring undernourished from preconception into the first 30 days of gestation, cortisol response is suppressed following arginine vasopressin (AVP) and corticotrophin-releasing hormone (CRH) stimulation [27]. Conversely, female offspring restricted from preconception only until the first 7 days of gestation has a greater cortisol response to stress [28]. Elevated basal cortisol and greater cortisol response to stress have been found in offspring following undernutrition strictly in the first 30 days of gestation [26, 91].

Alterations to the growth and function of the HPA axis that persist postnatally following periconceptional undernutrition are potentially mediated by epigenetic modifications to molecules implicated in adrenal growth and steroidogenesis. Specifically, insulin-like growth factor 2 (IGF-2) and its receptor IGF-2R are parentally imprinted genes involved in these processes [92]. Zhang et al. [28] nutritionally restricted normal and overnourished ewes from one month prior to conception until one-week gestation. At this point, embryos were transferred to normal weight “host” ewes that were maintained on a regular diet. Offspring exposed to undernutrition had significantly larger adrenal glands and a greater cortisol response to stress at 4 months of age. These changes were associated with reduced adrenal expression of IGF-2 mRNA, mediated by loss of methylation in the* IGF2/H19* differentially methylated region (DMR) [28]. Interestingly, the cohorts exposed to the Dutch Famine during the periconceptional period also exhibit less DNA methylation of* IGF2* as adults compared to their unexposed sex-matched siblings [81].

Thus, the periconceptional period is an important stage in which nutrition status may influence programming of the HPA axis.

### 8.2. Periconceptional Undernutrition and Offspring Growth, Body Composition, and Energy Regulating Pathways

Regulation of fetal growth and postnatal body composition appear to be at least partially programmed by the maternal reproductive environment in very early development.

#### 8.2.1. Disrupted Gestational Growth Patterns

Periconceptional undernutrition alters the normal relationships of early gestational weight gain with placental and fetal growth [29]. Following periconceptional restriction, the growth of the fetoplacental unit is uncoupled from maternal weight gain. Additionally, there is a significant retardation of growth in response to an applied stress in late gestation, with the most pronounced effects in fetuses exposed to undernutrition strictly before conception [30]. Following undernutrition strictly before conception, offspring gain more weight in the first 12 weeks of life than periconceptionally restricted and control offspring [42]. A similar pattern of accelerated postnatal growth compared to controls was found in rat offspring exposed to undernutrition in the preimplantation period only [93]. This is associated with significant changes to blastocyst development during this time. This period is therefore likely to play an important role in determining fetal growth trajectory and the stimuli that alter its course.

#### 8.2.2. Increased Adult Adiposity

In male offspring only, periconceptional undernutrition increases percent fat mass, restricts linear growth, and leads to smaller relative organ weights in adult sheep [33]. This is despite similar birth weight, postnatal growth trajectory, and adult total mass to controls. Corroborating this is a study that found greater proportional fat mass in sheep conceived as twins, but with one terminated early in gestation [94]. It therefore appears that relative adiposity is determined very early in development. This proportional increase in fat has important consequences for adult health. Greater percent fat mass in the context of normal total mass is termed normal weight obesity. In humans, there is a linear relationship between body fat and metabolic syndrome in individuals with normal BMI, and body fat is an independent risk factor for cardiovascular mortality in women [95].

#### 8.2.3. Altered Regulation of Energy Intake and Expenditure

Alterations to the HPA axis following periconceptional undernutrition are not limited to adrenal gland structure and function. The hypothalamus is an important locus of control in energy homeostasis, particularly via feeding and activity drives. Persistent epigenetic modification of the hypothalamic proopiomelanocortin (POMC) and glucocorticoid receptor (GR) genes in late gestation offspring exposed to periconceptional undernutrition is likely to cause abnormal feeding regulation, as well as energy metabolism and expenditure in later life [31, 96]. Offspring exposed to periconceptional undernutrition has significantly lower voluntary activity levels as adults, with males demonstrating the lowest activity [34]. Thus, the periconceptional programming of adult disease may be mediated by inherent reductions in energy expenditure drives in addition to dysfunctional energy metabolism and storage.

### 8.3. Periconceptional Undernutrition and the Glucose-Insulin Axis

A potential pathway of altered fetal growth following periconceptional undernutrition is glucose metabolism. While there is evidence for the influence of late gestational nutritional status on later-life glucose metabolism [97], the periconception period also appears to be of importance.

#### 8.3.1. Altered Gestational Glucose Exposure and Metabolism

Insulin resistance is a normal adaptation to pregnancy, allowing for greater availability of glucose to the fetus [98]. Preconception and periconception undernutrition abolish maternal insulin resistance adaptations seen in control pregnancies in ewes [35]. Fetal growth is therefore dictated by an inverse relationship with maternal insulin sensitivity, and this is most pronounced when undernutrition was limited to preconception only [35].

Altered insulin response to glucose is evident in late gestation [36], and at 10 weeks postnatally [38], with greater insulin response following glucose challenge compared to controls [36]. Preimplantation, but not periconceptional undernutrition, results in higher circulating insulin in the late gestation fetus, marking the first few days of gestation as critical in this system [37]. Exaggerated insulin response in early life is believed to be suggestive of early pancreatic maturation or altered pancreatic beta cell function [36, 38].

#### 8.3.2. Impaired Adult Glucose Tolerance

In adult offspring, periconceptional undernutrition in normal weight ewes results in impaired glucose tolerance [40]. This is demonstrated by increased plasma glucose and a blunted insulin response to a glucose tolerance test at 10 months of age. These parameters indicate that reduced glucose clearance as a result of dysfunctional insulin secretion is the underlying mechanism modified by undernutrition. A rat model using isocaloric protein restriction in the periconception period also resulted in higher blood glucose and cholesterol in adult offspring [99].

#### 8.3.3. Epigenetic Modification of Insulin-Signalling Molecules

Epigenetic modification of hepatic insulin-signalling molecules may explain these fetal and postnatal differences in glucose metabolism following periconceptional undernutrition. Nicholas et al. [39] found a reduced subset of these molecules (IRS1, PDK1, and aPKC) in 4-month-old lambs. Low levels of these molecules are likely to result in an impaired hepatic response to insulin in the context of nutrient abundance. Predisposition for later-life insulin resistance is therefore more likely in animals with these changes [39]. There are also epigenetic modifications to visceral fat depots likely to impair thermogenic capacity and insulin sensitivity of adipose tissue in postnatal life [37]. Thus, altered energy metabolism that may be protective in a nutritionally restricted fetal environment predisposes the postnatal offspring to metabolic disease when faced with an energy-abundant environment.

### 8.4. Cardiovascular Adaptation to Periconceptional Undernutrition

In addition to maladaptive metabolism, adult disease is largely influenced by impaired cardiovascular function. Hypertension is an important component of the metabolic syndrome and is strongly related to cardiovascular disease [100].

Cardiovascular adaptations to periconceptional undernutrition are apparent in late gestation, demonstrated by increased fetal blood pressure [41]. In twins, rate-pressure product is also higher than controls, and blood pressure is positively correlated with ACTH levels [41]. This is an early indication that HPA axis changes may extend to vascular regulation following periconceptional restriction.

Torrens and colleagues [42] found that pre- and periconceptional undernutrition result in vascular reactivity changes in adult offspring that are vascular bed dependent. Both pre- and periconceptional undernutrition are associated with enhanced vasoconstriction of the coronary arteries and endothelial dysfunction in femoral resistance arteries. This is relevant to the Dutch Famine data finding higher rates of coronary artery disease among people exposed to famine in early gestation [68]. Interestingly, vasoconstriction responses are greater in multiple vascular beds in periconception versus preconception undernutrition, identifying the preimplantation period as potentially more important in the programming of vasoconstriction response [42].

While there are well-established links between overtly elevated basal cortisol and hypertension [101], there is also evidence that hypertension can be associated with HPA axis dysfunction in the absence of deranged basal cortisol levels [102]. Human subjects with hypertension have a greater cortisol response to CRH challenge, despite having similar baseline cortisol to controls [103]. It is therefore conceivable that altered HPA axis findings in periconceptionally restricted offspring may help to explain cardiovascular dysfunction.

## 9. Conclusions, Implications, and Recommendations

Periconceptional undernutrition is associated with a number of differences in fetal and postnatal measures, many of which appear to be related to the HPA axis. Some of these modifications may act as compensation in the immediate period to protect and prepare the offspring for an adverse environment. Further development in an environment with abundant energy availability may then predispose the offspring to metabolic and cardiovascular disease.

Further clarification is required as to which periods of development are most important and therefore most sensitive to undernutrition. With respect to human pregnancy, which is rarely diagnosed until several weeks gestation, it is likely that undernutrition at any point in periconception will have consequences.

The mechanisms by which the nutrition insult results in long-term changes in the offspring are still unclear. Ongoing research into the role of epigenetics will probably contribute to further understanding of programming response to adversity.

Finally, human studies are lacking in the field of weight loss prior to pregnancy. Given the animal data, both the benefits and risks of losing weight prior to conception need to be identified in clinical populations. This is necessary to design evidence-based practice guidelines for managing obesity in the obstetric population.

## Figures and Tables

**Table 1:** Summary of animal study findings associated with periconception and early gestation maternal weight loss.

HPA axis development and function	
(i) Altered placental 11betaHSD activity and cortisol : cortisone in fetal circulation [20, 21]	
(ii) Accelerated activation of fetal HPA axis in late gestation [22–25]	
(iii) Preterm birth [23–25]	
(iv) Enhanced HPA axis response to CRH stimulation at 2 months [26]	
(v) Blunted cortisol response to CRH and AVP stimulation in adult offspring [27]	
(vi) Increased adrenal gland size in males and females and greater stress response in adult female offspring accompanied by epigenetic changes to adrenal *IGF2/H19* gene [28]	

Growth, body composition, and energy regulating pathways	
(i) Altered relationships between maternal weight and fetoplacental growth in early pregnancy [29]	
(ii) Altered fetal growth response to late gestation stressors [30]	
(iii) Epigenetic changes in POMC and GR genes in fetal hypothalamus [31]	
(iv) Reduced fat mass in offspring of overweight ewes [32]	
(v) Greater percent fat mass and smaller relative heart, lungs, and adrenals in male offspring [33]	
(vi) Decreased voluntary physical activity in adult offspring [34]	

Glucose-insulin axis	
(i) Impaired pregnancy insulin resistance [35]	
(ii) Increased fetal insulin response to glucose in late gestation [36]	
(iii) Altered thermogenic, insulin, and fatty acid oxidation signalling in fetal perirenal fat depot [37]	
(iv) Altered glucose-insulin metabolism in adult males [38]	
(v) Epigenetic modification of hepatic insulin-signalling molecules [39]	
(vi) Impaired glucose tolerance in adult offspring [40]	

Cardiovascular function	
(i) Increased late gestation fetal blood pressure [41]	
(ii) Enhanced vasoconstriction in adult female coronary arteries and endothelial dysfunction in femoral resistance vessels [42]	
